# Easy Handling and Cost-Efficient Processing of a Tb^3+^-MOF: The Emissive Capacity of the Membrane-Immobilized Material, Water Vapour Adsorption and Proton Conductivity

**DOI:** 10.3390/nano12244380

**Published:** 2022-12-08

**Authors:** Estitxu Echenique-Errandonea, Ricardo Faria Mendes, Flávio Figueira, Paula Barbosa, Sara Rojas, Duane Choquesillo-Lazarte, Javier Cepeda, Duarte Ananias, Filipe Figueiredo, Filipe A. Almeida Paz, Antonio Rodríguez-Diéguez, José Manuel Seco

**Affiliations:** 1 Departamento de Química Aplicada, Facultad de Química, Universidad del País Vasco UPV/EHU, Paseo Manuel Lardizabal, Nᵒ 3, 20018 Donostia-San Sebastian, Spain; 2 Department of Chemistry, CICECO–Aveiro Institute of Materials, University of Aveiro, 3810-193 Aveiro, Portugal; 3 Department of Physics, CICECO–Aveiro Institute of Materials, University of Aveiro, 3810-193 Aveiro, Portugal; 4 Departamento de Química Inorgánica, Facultad de Ciencias, Universidad de Granada, Av. Fuentenueva S/N, 18071 Granada, Spain; 5 Laboratorio de Estudios Cristalográficos, IACT, CSIC-UGR, Av. Las Palmeras N◦ 4, Armilla, 18100 Granada, Spain

**Keywords:** metal–organic frameworks, processing and shaping, emission capacity, water adsorption, ionic conductivity

## Abstract

The development of convenient, non-complicated, and cost-efficient processing techniques for packing low-density MOF powders for industry implementation is essential nowadays. To increase MOFs’ availability in industrial settings, we propose the synthesis of a novel 3D Tb-MOF (**1**) and a simple and non-expensive method for its immobilization in the form of pellets and membranes in polymethacrylate (PMMA) and polysulphone (PSF). The photoluminescent properties of the processed materials were investigated. To simulate industrial conditions, stability towards temperature and humidity have been explored in the pelletized material. Water-adsorption studies have been carried out in bulk and processed materials, and because of the considerable capacity to adsorb water, proton-conduction studies have been investigated for **1**.

## 1. Introduction

The development of crystalline porous materials with long-range ordered structures which offer structural diversity and tunability has been of potential interest since applications in many aspects of everyday life and industrial applications can be found [1,2,3,4,5,6,7]. Still, several technical barriers must be exceeded for these materials to be implemented in the industry [8]. Among others, we can mention the availability at large-scale, which not only depends on reactants but on the whole process production costs, MOFs’ physical properties maintenance when manufacturing at a high scale, and the importance of formulation for fine-tuning the physical properties of MOFs when shaping for a particular application, in addition to the performance of the final material under realistic operating industrial conditions such as moisture, pressure or temperature [9].

MOFs are typically isolated as insoluble and low-density loose powders that are often difficult to handle and can be problematic to incorporate into devices for the industry because they can easily blow away and contaminate pipes [10]. Therefore, shaping/processing MOFs is mandatory for using them in real industrial applications. Generally, the processing or shaping technique selection depends on the MOF synthetic procedure and textural properties [10,11].

After carrying out MOFs processing, the mechanical strength and resistance are expected to be improved compared to the bulk, non-processed material. Additionally, the shaping must be cost-effective [11]. Even if the shaping of a MOF for specific applications is still in its infancy, great efforts need to be dedicated to the rational study of this process to access its real commercial applications [10].

Monoliths, beads, and pellets are conferred as suitable processed materials with maximized bulk density and minimized wasted space [11]. Among the mentioned transformed materials, the pelletization technique is the most common method for densifying MOFs by applying pressure. This process encounters, however, several disadvantages, such as excessive pressure that can crush the MOFs structure and reduce mechanical stability, as well as the use of binders (generally added to improve cohesion), which can dilute the porous powder and cause pore blockage [10].

Another interesting approach for transforming materials without external pressure is assembling MOFs into polymeric membranes, giving rise to mixed-matrix membranes (MMMs) [12]. This strategy is appealing from an industrial perspective because membranes have low production cost and exhibit relatively high mechanical properties such as flexibility, softness, and thermal and chemical stability, amongst others [12,13,14].

From a practical point of view, once processed, shaped materials are required, apart from keeping structural robustness, to be stable under humidity, temperature, or pressure operating conditions [9]. Thus, testing shaped materials towards these variables becomes highly important for optimal performance in industrial implementation.

In addition, MOFs have found their space in proton conduction, relying on their intrinsic large surface areas and long-range ordered and porous cavities, where proton carriers can be incorporated as guest molecules (e.g., water, imidazole, ammonium cations, involatile acids, etc) or by the introduction of acidic groups (e.g., −COOH, −PO_3_H, −SO_3_H, and −OH) into the framework backbone, enabling control over conductivity at the molecular level [15]. Thus, in proton-conductive MOFs, the complex hydrogen-bond network offers a suitable pathway for proton migration by either the “vehicle” or the “Grotthus” mechanisms. In the former, proton-diffusion occurs enabled by water molecules that act as vehicles, and in the “Grotthus mechanism”, the proton hops from one site to another through H-bonding with sequential molecular rotational reorientation [16]. Therefore, the hydrophilic character of the MOF is of great importance and plays a key role. In this regard, hydrophilic MOFs display strong water affinity and can absorb a large amount of water molecules even under low humidity conditions. Additionally, acidic protons or metal-coordinated water molecules facilitate the hydrogen bonding formation path inside the pores, promoting proton conduction [17].

The aforementioned property is directly correlated to the water-adsorption capacity of the MOF. Among water-adsorption mechanisms, three types are described: vapour adsorption on the metallic cluster (chemisorption), layer adsorption (this process is reversible and is correlated to physisorption) and capillary condensation (this process is reversible) [17]. Since the first report on proton conduction by a copper-based two-dimensional coordination polymer in 1979 [18], several examples have been published [19,20]. Nonetheless, the design of MOFs capable of adsorption and desorption of a large amount of water molecules within a narrow relative humidity range remains a challenge [15,16].

Having these challenges in mind, in this work, we propose easy handling techniques for shaping compound **1**, a Tb^3+^-based MOF isotypical to our previously reported Eu-MOFs [21] formulated as {[Tb_5_L_6_(OH)_3_(H_2_O)_3_]·5DMF]}_n_. This was accomplished by processing **1** into pellets and membranes and by studying the shaped materials’ performance against temperature and moisture conditions to simulate possible industrial operating conditions. Additionally, as proof of principle, the photoluminescence properties of **1** incorporated into the PMMA membrane were investigated, in line with the work performed by Dechnik et al., who described the first luminescent MOF mixed-matrix [22]. Motivated by the relatively high vapor-adsorption capacity and structure stability of **1**, the proton conductivity was further studied [15,16].

## 2. Materials and Methods

### 2.1. Preparation of Complexes

All chemicals were of reagent grade and were used as commercially obtained. For all tested synthetic methods, terbium(III) nitrate hydrate (99.9%, Alfa Aesar, Haverhill, MA, USA) was used as metallic precursors, and 3-amino-4-hydroxybenzoic acid ligand (H_2_L, C_7_H_7_NO_3_, 97% of purity) was purchased from Fluorochem. Methyl methacrylate polymer (PMMA) was obtained from TCI, Polysulphone (PSF) (average Mw ~35,000 by LS, average Mn ~16,000 by MO) from Aldrich, and dichloromethane (CH_2_Cl_2_, pure) from Sigma-Aldrich.

### 2.2. Synthesis of {[Tb_5_L_6_(OH)_3_(H_2_O)_3_]·5DMF}_n_

Previous work has reported the synthetic procedure to obtain a single crystal and the scale-up synthesis of **1** [21].

**Figure 1:**
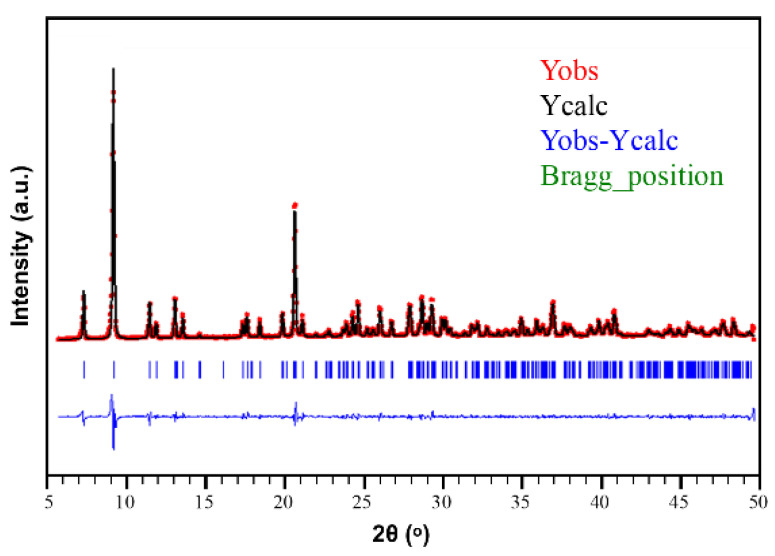
Figure of the pattern matching analysis and experimental PXRD for compound **1**.

Briefly, 0.010 g (0.0625 mmol) of the 3-amino-4-hydroxybenzoic acid organic linker was dissolved in 0.2 mL of DMF containing 10 μL of Et_3_N (0.072 mmol). A total of (0.0189 g) 0.0434 mmol of the Tb(NO_3_)_3_·*n*H_2_O (where *n* = 5 or 6) was dissolved into 0.8 mL of distilled water in a separate vial. Once dissolved, 0.8 mL of H_2_O was added to the ligand solution and 0.2 mL of DMF to the metal solution. The metal solution was added dropwise to the ligand solution under constant magnetic stirring. The resulting brownish-yellow solution was poured into a screw-capped vial (6 mL) and placed in the oven at 100 °C for 2 h to give rise to hexagonal-shaped single crystals. For the scale-up synthesis, a microwave procedure was employed, weighing 0.2 g (1.2 mmol) of 3-amino-4-hydroxybenzoic acid ligand and dissolving in a solvent mixture of DMF/H_2_O (1.2 mL/3.0 mL) containing 200 µL of Et_3_N (1.44 mmol). To this solution, a solution containing 0.38 g (0.868 mmol) Tb(NO_3_)_3_·*n*H_2_O dissolved in 1.8 mL water was added dropwise. The brownish solution was placed in a microwave and heated at 100 °C for two hours to obtain bulk crystalline powder (yield ~25%). In both synthetic procedures, PXRD confirmed the purity of the product (Figure 1, Table S1).

### 2.3. MOF Processing into Pellets and Membranes

#### 2.3.1. Membrane’s Preparation and Characterization

The integration of **1** into membranes employed two polymeric matrices: polysulphone (PSF) and polymethylmethacrylate (PMMA).

The membrane preparation conditions for both matrices were optimized by testing different polymer-to-MOF ratios to achieve a material with balanced mechanical stability and homogeneity of membrane coverage. This optimization is required because an excessive MOF/polymer ratio diminishes the mechanical strength of the membrane, which becomes more fragile. Low ratios typically lead to pockets in the material (areas not covered with the MOF). Therefore, an optimized equilibrium is of great importance to obtain a material with the best properties.

**Scheme 1:**
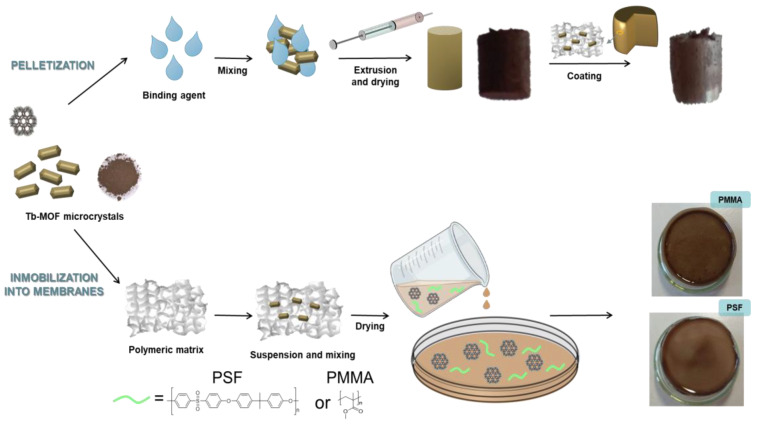
Representation of the pelletization and membrane immobilization of compound **1**.

After several optimization attempts, the optimal membrane conditions were established to be 75 mg MOF in 400 mg polymer. This way, the mechanical stability and homogeneity of the processed material could be compared as the same synthetic ratio has been employed in both polymeric matrices. The membrane synthesis was performed according to the following procedure (Scheme 1): 400 mg of PSF were weighed and dissolved in 5 mL of CH_2_Cl_2_; to this dense solution, 75 mg of **1** were added and left stirring for 30 min. The remaining viscous solution was then cast in a glass petri dish and left unstirred at ambient conditions until complete evaporation of the solvents. In the case of PMMA, the same solvent volume was measured, and 400 mg of polymethylmethacrylate was added. After complete dissolution, 75 mg of **1** was added, and the suspension was stirred for 30 min. The succeeding steps were followed as previously described for PSF-based membranes (Figure S2).

MOF incorporation into the polymeric membrane was accessed by powder X-ray diffraction and SEM/EDS analysis.

Additionally, incorporation is immediately visible to the naked eye as pure PMMA and PSF are transparent, while MOF-containing membranes have dark brown colour (Figure S2). Moreover, SEM/EDS mapping of the surface and cross-section analysis allowed confirmation that **1** was uniformly distributed throughout the polymeric membrane (Figure S3).

#### 2.3.2. Pellets Preparation and Characterization

A homemade apparatus composed of a syringe was used to extrude samples in the form of pellets (Figure S1), using water as the binding agent. The procedure starts by preparing a mixture of 100 mg of **1** with 100 µL of water to form a malleable paste, and extruding in the form of a cylinder 5 mm in diameter and 10 mm in length. The binding water was eliminated by drying at 125 °C for 2 h in an oven/etc., to obtain the final pelletized material (Scheme 1). With the aim of enhancing resistance, some of pellets were coated with polymer; in this case, polysulphone was used for the purpose. For polymer coating, the pellet was submerged for 2 s in an already prepared polymer solution of CH_2_Cl_2_ containing 300 mg of polysulphone. Once the dichloromethane was completely evaporated and the coating dried, the pellet reached its final resistance.

## 3. Results

### 3.1. Crystal Structure Details

**Figure 2:**
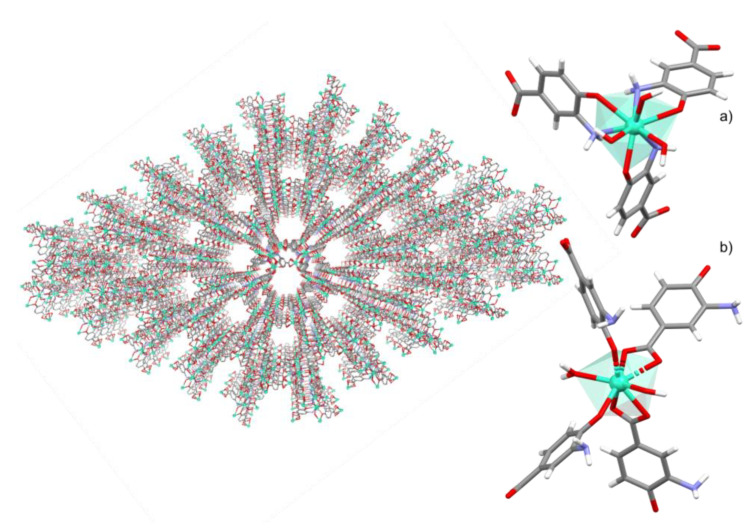
Packed structure of compound **1** (left) and (**a**) nine- and (**b**) eight-coordinated Tb environments found in the asymmetric unit of Tb-MOF.

Compound **1** exhibits a three-dimensional MOF structure that crystallizes in the *P*6_3_/*m* space group (Figure 2, Tables S2 and S3). The asymmetric unit is comprised of two crystallographically independent Tb^3+^ ions displaying nine- and eight-coordinated environments that, according to SHAPE [23], build spherical tricapped trigonal prism (TCTPR-9) and square antiprism (SAPR-8) polyhedra, respectively (Tables S4 and S5). A deprotonated ligand and a coordinated water molecule, as well as a hydroxyl group which plays the role of connecting adjacent metallic centres, complement the asymmetric unit.

The nine-coordinated environment is composed of three hydroxyl oxygen atoms and three amino groups of 3-amino-4-hydroxybenzoate ligands, in addition to three oxygens belonging to hydroxyl bridges, whereas the metal centre, having a lower coordination environment, is composed of six oxygens from carboxylate groups, two phenoxide oxygens, and an additional oxygen belonging to a coordinated water molecule and to a hydroxyl connecting group.

The crystal structure contains, along the crystallographic *c* axis microchannels where crystallization solvent molecules are embedded, in this case, solvent DMF molecules. The connectivity was evaluated by TOPOS software [24], exhibiting ***acs*** net type with **4^9^·6^6^** point symbol where pentanuclear nodes are connected by 6-connected unimodal type net. The synthesized three-dimensional open MOF contains large microchannels of appropriate pore size of (7.4 Å), which allows the effective inclusion of solvent molecules. The aforementioned channels correspond to ca. 19% of the unit cell (according to the geometrical calculation of the pore volume with the PLATON-v1.18 program), and host crystallization DMF molecules.

### 3.2. Pellets and Membranes Stability Studies

To incorporate MOFs into devices, bulk material shaping is of great importance [11]. To explore processing techniques, **1** was transformed into pellets and membranes. Once **1** was supported into pellets and membranes, the stability of these processed materials was tested against moisture and temperature. For that purpose, materials were placed for 72 h in a desiccator containing a K_2_SO_4_ saturated solution, which simulated 98% relative humidity (RH) [25]. Afterwards, materials were characterised by PXRD.

#### 3.2.1. Moisture Stability

**Figure 3:**
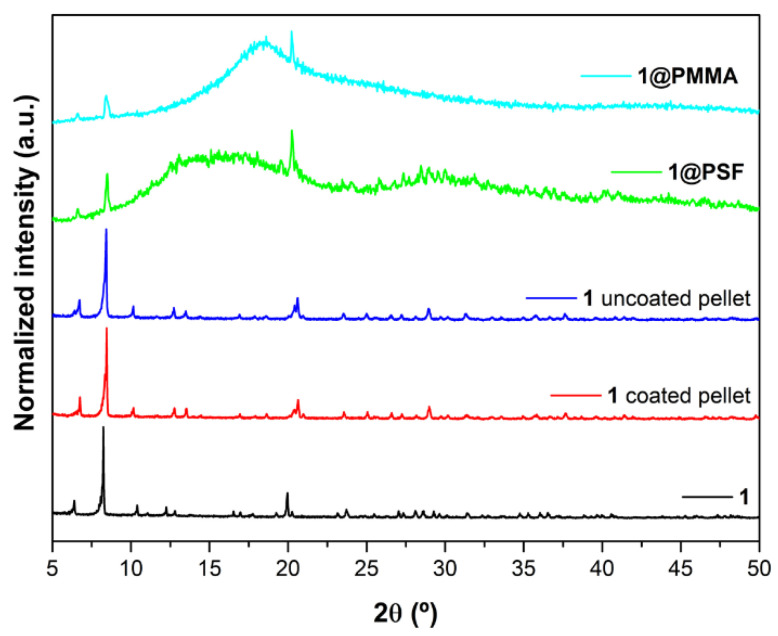
Powder X-ray diffractograms of the studied materials, as-synthesized and after processing into pellets and membranes.

PXRD results show that **1** and the corresponding pellets or membranes are stable even with prolonged exposure to elevated RH (72 h at 98% RH) (Figure 3). Due to the amorphous nature of the polymer, only the main intense peaks of **1** stand over the amorphous background in polysulphone (PSF) based membranes PXRD.

#### 3.2.2. Temperature Stability

**Figure 4:**
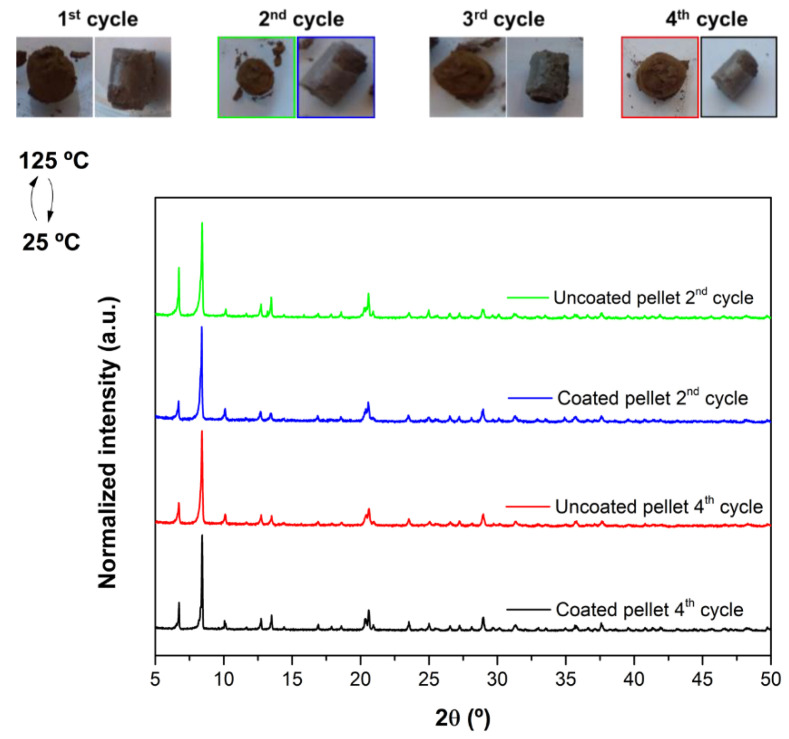
Powder X-ray diffractograms of the studied materials, as-synthesized and after processing into pellets.

After the humidity tests, the pellets were treated using temperature cycles. Four heating and cooling down cycles were performed, and materials were characterised by PXRD analysis (Figure 4). Uncoated pellets and polysulphone-coated ones were first tested against 98% relative humidity (RH) for 72 h and later tested for their thermal stability. For this purpose, temperature cycles of heating to 125 °C and cooling down to ambient temperature were carried out. After each cycle, a photograph of both pellets (coated and uncoated) was taken to check their integrity. Subsequently, after the second and fourth cycles in coated and uncoated pellets, PXRD analysis was performed to assess the overall stability (Figure 4).

### 3.3. Water Vapour Adsorption Studies

**Figure 5:**
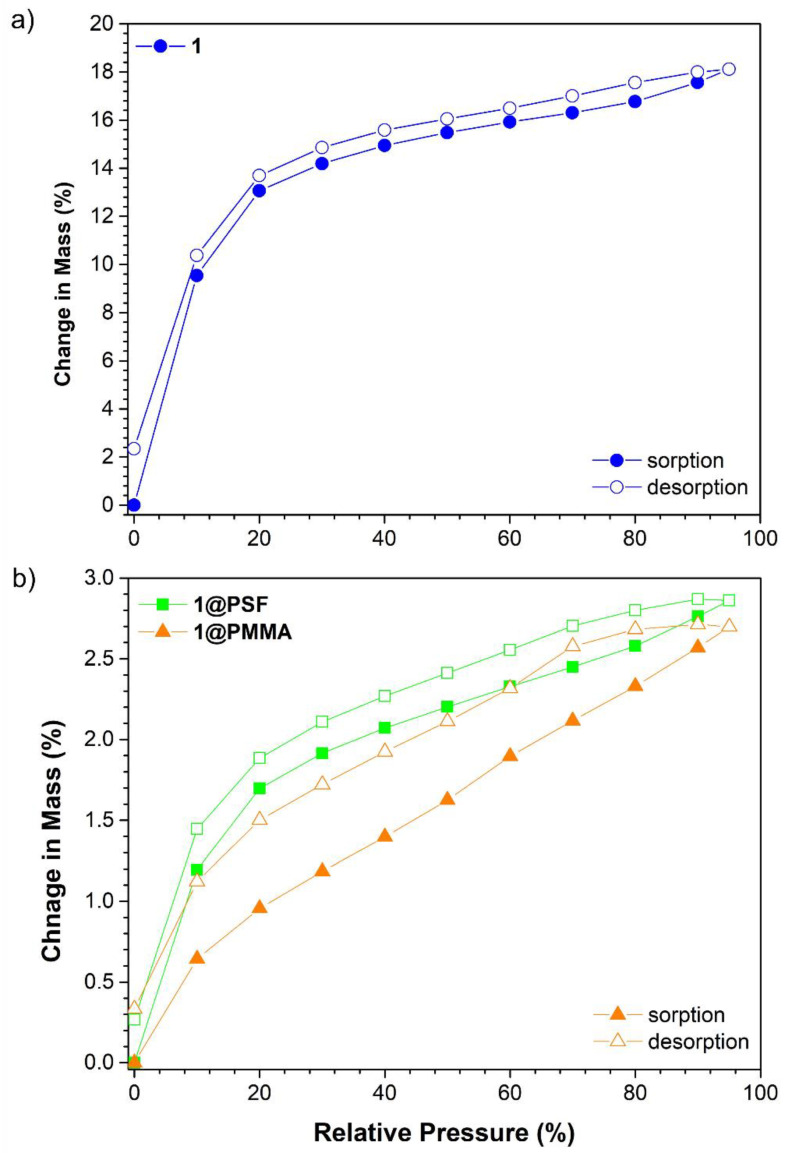
Water vapour adsorption (solid symbols) and desorption (open symbols) isotherm curves for (**a**) compound **1**, and (**b**) **1@PSF** and **1@PMMA** membranes.

The gravimetric water vapor sorption kinetic measurements of **1** and composite membranes **1@PSF** and **1@PMMA** (Figure 5) were performed using a Dynamic Vapor Sorption (DVS) system at 25 °C over a wide range of relative humidity (0% to 95% RH). The isotherm of **1** displays a Type I shape curve, typical of microporous materials such as MOFs, although the slight increase in water mass above 80% RH (seen as a hint of a TypebII isotherm) can be seen as an indication of a minor contribution of multilayer adsorption on the external surface of the particles. The overall increase in mass of **1** is quite substantial, reaching ca. 18 wt.% at *P*/*P*_0_ = 95%. There is a slight, but obvious, hysteresis between the adsorption and desorption, which extends to the low-pressure range, indicating the retention of water molecules within the MOF framework. This irreversible water uptake may be related to the swelling of the MOF structure or a chemical interaction with the water molecules.

The isotherm of the **1@PSF** membrane displays a similar shape to that of **1**, in turn very different from the Type III observed for pure PSF [26], and thus suggesting that the overall mass change is determined by the fraction of **1** in the membrane. As expected, **1@PSF** adsorbs much less water than **1**, reaching a maximum of 2.86 *wt*.% at *P*/*P*_0_ = 95%. Assuming that pure PSF adsorption is around ca. 0.8 wt.% of water, also at *P*/*P*_0_ = 95% [26], one can crudely estimate the weight fraction of **1** in PSF to be 12%, which is in reasonable agreement with the nominal composition.

**1@PMMA** absorbs nearly the same amount of water near saturation (2.70 wt.% at *P*/*P*_0_ = 95%) than **1@PSF**, but the shape of the isotherm for low RH indicates a much smaller fraction of the micropores accessible for adsorption. The poor affinity of PMMA for water (adsorbs ca. 0.06 wt.% at *P*/*P*_0_ = 95% [27]) could explain this behaviour.

### 3.4. Electrical Conductivity

Motivated by the relative high-water vapour uptake, we decided to measure the ionic conductivity of the pelletized sample of compound **1** and of composite membranes based on PSF and PMMA, respectively. One should recall that the pellet employed for the electrical measurements was obtained after pressing pure compound **1** in the form of powder in a uniaxial press at 800 MPa. Conductivity measurements were carried out in pelletized samples of **1** with increasing temperature at each RH (1st cycle) and cooling down (2nd cycle). As expected for water-mediated proton conducting MOFs, proton conductivity increases with the augmentation of humidity.

**Figure 6:**
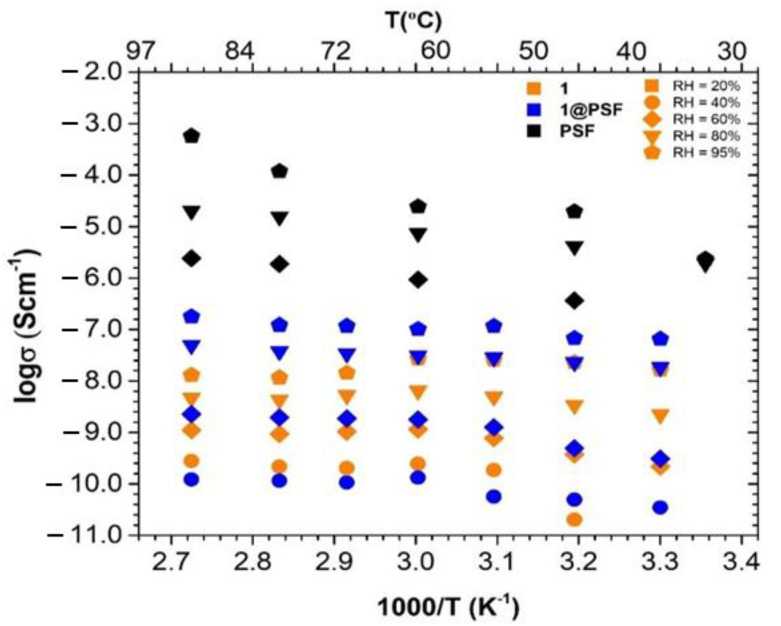
Arrhenius plots for the ionic conductivity of compound **1**, **1@PSF** membrane and pristine PSF in-plane conductivity measured under variable RH and temperature.

The Arrhenius plots of the ionic conductivity of **1** and **1@PSF** membrane, measured under the variable temperature (40–94 °C) and relative humidity (20–95% RH), are presented in Figure 6. The ionic conductivity results of the **1@PMMA** membrane are not represented in Figure 6, since the electrical resistance of these samples was higher than 10 MΩ and, thus, impossible to determine due to the limitations of the LCR meter. According to the data in Figure 6, Figures S5 and S6 the ionic conductivity of the materials increases with temperature and RH, indicating that the conductivity of the material is provided by the protons of the water molecules adsorbed in the porous MOF structure. In the case of **1**, at temperatures higher than 60 °C and RH ≥ 40%, the conductivity started to decrease, possibility due to some sort of phase transition or to a degradation in particle connectivity due to the presence of an increasing number of water molecules. The maximum ionic conductivity observed for **1** was 3.2 × 10^−8^ Scm^−1^ at 60 °C and 95% RH, which is a relatively modest value when compared with the conductivity of other 3D carboxylate-based MOFs, which ranges from 10^−6^ to 10^−2^ Scm^−1^ (Table S7) [28,29,30,31,32,33,34,35,36,37]. From DVS data, one would expect higher conductivity values, which may be explained by (i) some degradation of the MOF bulk framework during ionic conductivity measurements, and (ii) the pelletization of the MOF, which decreases the available surface area. The addition of compound **1** to the PSF membrane leads to conductivities in the range of 10^−11^ to 10^−7^ Scm^−1^ (from 40 to 95% RH), which are about three orders of magnitude lower when compared with pristine PSF membrane (Figure 6). The conductivity of neat PSF assessed by in-plane configuration was also measured (Figure 6), and the maximum conductivity obtained was 5.75 × 10^−4^ Scm^−1^ at 94 °C and 98% RH, which is in line with the through-plane conductivity value reported by K. Deepa et al. (1.16 × 10^−4^ Scm^−1^ at 23 °C [38]. These results suggest that compound 1 is a poor ionic conductor, probably because of the lack of functional media that can promote proton transport between the ionic groups. Moreover, the presence of the MOF in the PSF matrix seems to contribute to a slight increase of the polymer conductivity at low RH (20%). On the other side, PSF seems to increase the stability of the MOF, since the decrease of the conductivity with temperature is no longer observed.

### 3.5. Photoluminescent Properties

Lanthanide-based MOFs have promising luminescent applications because of the characteristic narrow emission lines of the trivalent lanthanide cations, ranging from the UV to the near IR, and because of the superior capability of the organic ligands to capture UV light. With the aim of further improving the UV light capture, and approaching the implementation into luminescent devices, we decide to incorporate a polymeric membrane, the Tb^3+^-MOF, recently reported by our group [39]. For this purpose, we carefully selected PMMA as the polymeric matrix to avoid any possible interference between the Tb^3+^-MOF and the polymeric intrinsic emissions. In addition, with the aim of exploring ligand emission, we selected isotypical Y^3+^ MOF [39], namely **2**.

**Figure 7:**
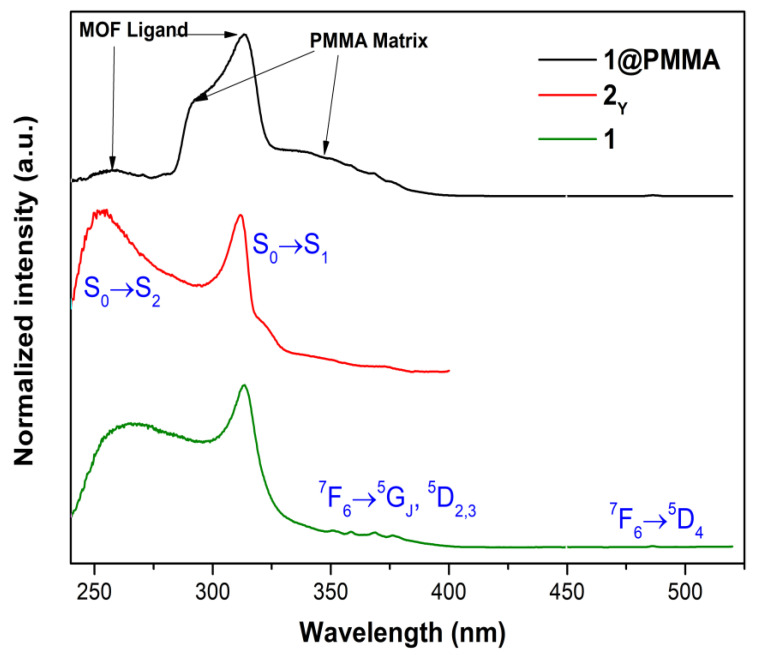
12 K excitation spectra of **1@PMMA** (black; λ_Em_. = 544 nm), the isotypical Y^3+^-based MOF **2_Y_** (red; λ_Em_. = 410 nm), and **1** pristine Tb^3+^ MOF) (green; λ_Em_. = 544 nm).

The excitation spectrum of compound **1@PMMA,** recorded at 12 K, with the detection fixed on the Tb^3+^ emission at 544 nm (Figure 7), shows the UV bands attributed to the ligand MOF and to the PMMA polymeric matrix (Figure S4). The positions of the S_0_→S_2_ and S_0_→S_1_ peaks at ca. 258 and 313 nm, respectively, are attributed to the ligand MOF because they are identical to those observed for the isotypical Y^3+^ and Tb^3+^ pristine MOFs [39] (Figure S5). The additional shoulders, at ca. 290 nm and from 330 to 380 nm, should be attributed to the PMMA matrix interacting with the surface of the MOF particles. The intensity decreases of the intra 4f Tb^3+^ excitation lines for **1@PMMA** result from the dilution of the Tb^3+^ MOF in the polymeric membrane.

**Figure 8:**
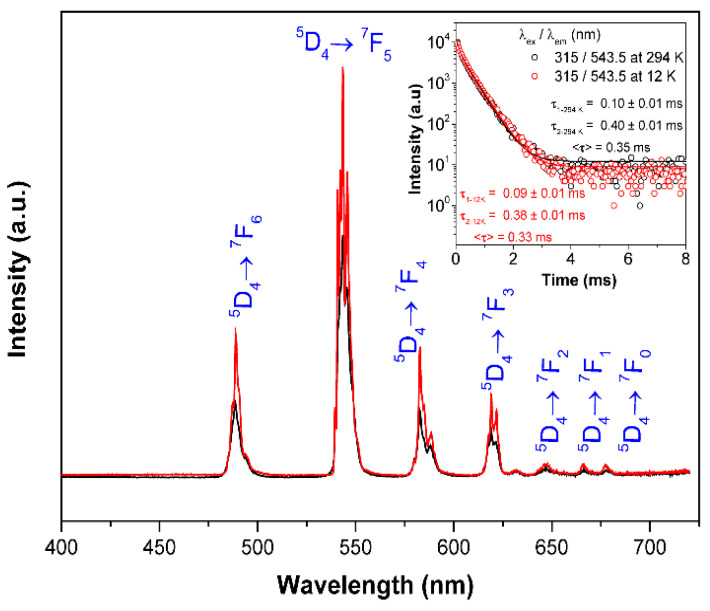
Emission spectra of 1@PMMA recorded at 294 K (black) and 12 K (red), with the excitation fixed at 315 nm. Inset: 5D4 decay curves of 1@PMMA acquired at 294 K (black) and 12 K (red), monitoring the Tb3+ emission at 544.5 nm with the excitation fixed at 315 nm. The solid lines are the best fits using second order exponential decay function; y = y_0_ + A_1_·exp(−x/$\tau_{1}$) + A_2_·exp(−x/$\tau_{2}$ ) (r^2^ > 0.999).

On its part, upon excitation at 315 nm, the MOF-loaded polymeric membrane displays an appreciable green emission from Tb^3+^, both at 294 and at 12 K as shown in Figure 8. The observed sharp emission lines are assigned to the characteristic Tb^3+ 5^D_4_→^7^F_0-6_ transitions. As expected, the emission intensity increases with the decrease of temperature due to the extinction of the non-radiative relaxation pathways. The near absence of the ligand triplet emission, identified below at wavelengths ranging from 400 nm to 475 nm, indicates an efficient ligand-to-Tb^3+^ energy transfer process through the triplet excited state, commonly denominated the antenna effect.

The ^5^D_4_ decay curves were recorded at 294 and 12 K, fixing the excitation at 315 nm and monitoring the emission at 543.5 nm (Figure 8, inset). In both cases, the exponential decay curves are only properly fitted by a second order exponential function yielding two lifetimes with averaged lifetimes of $<\tau_{294\;K}>=0.350\;\mathrm{ms}$ and $\langle \tau_{12\;K}\rangle =0.326\;\mathrm{ms}$ (Table 1 and Table S6). This is in accordance with the presence of two distinct Tb^3+^ sites in the MOF structure.

**Figure 9:**
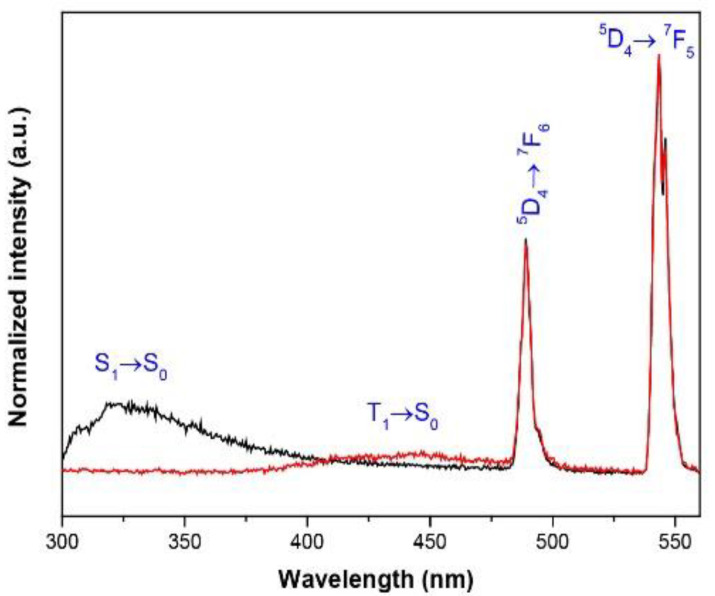
Time-resolved emission spectra of **1**@PMMA recorded at 12 K measured with 290 nm excitation. Black line: initial delay of 0.01 ms and integration time of 0.1 ms; red line: initial delay of 0.05 ms and integration time of 5 ms. Time-resolved emission spectra were not corrected for detection and optical spectral response of the spectrofluorometer.

Time-resolved emission spectra recorded at 12 K were excited at 290 nm and allowed to identify and differentiate ligand fluorescence (S_1_→S_0_ transition) and phosphorescence (T_1_→S_0_). The S_1_→S_0_ transition (fluorescence) has much faster time dependence than the T_1_→S_0_ transition (phosphorescence). The former transition is entirely suppressed by a time delay of only 0.05 ms (Figure 9). The strong suppression of the low-energy T_1_→S_0_ ligand emission indicates an efficient energy transfer from the triplet excited state to the Tb^3+^ excited levels. The zero-phonon energy level of the ligand phosphorescence emitting triplet states estimated at 390 nm (25,641 cm^−1^) is relatively close to the Tb^3+^ first excited state (^5^D_4_, 485 nm/20,619 cm^−1^) and, thus, justifies an efficient ligand-to-Tb^3+^ energy transfer.

## 4. Conclusions

Herein, we presented a non-complicated easy handling and cost-efficient method for the processing of MOFs into pellets and membranes, exhibiting in both cases adequate resistance and mechanical stability in the final shaped material towards high humidity and temperature cycling conditions.

The photoluminescent properties of the membrane supported with **1** have been investigated, proving that the terbium based-MOF can keep its emissive properties subsequently after processing into membranes; thus, this approximation offers a possible effective approach toward the implementation into devices of luminescent Ln-MOFs. Nonetheless, further experiments related to quantum yield may be required in order to determine emissive efficiency. Additionally, as a proof of concept, processed materials (pellets and membranes) have been tested against high humidity and temperature conditions, exhibiting relatively good resistance and performance under industrial operating conditions. Finally, motivated by the stability and resistance towards RH shaped materials, water adsorption isotherms have been collected, showing an 18% increment in mass at higher measured relative pressure, which corresponds to 95%.

In this line, as a possible application, proton-conduction has been tested. As expected for water-mediated proton conducting MOFs, proton conductivity increases with the augmentation of humidity, obtaining a maximum ionic conductivity value of 2.7 × 10^−8^ Scm^−1^ at 60 °C for **1**; comparing with the results in the literature, which exhibit conductivity values close to 1.1 × 10^−4^ Scm^−1^ for purely 3D carboxylate-based MOFs [28,29,30,31,32,33,34,35,36,37] (Table S7), **1** exhibits a relatively modest conductivity. From water adsorption (DSV) data, it would be expected to have higher conductivity values. However, these results can be explained by MOF degradation diminishing the overall available surface area by material processing.

## Figures and Tables

**Table 1:** 294 K and 12 K emission lifetime values and average lifetime fitted by a second order exponential decay function: y = y_0_ + A_1_·exp(−x/$\tau_{1}$ ) + A_2_·exp(−x/$\tau_{2}$).

Temperature (K)	λex/λem (nm)	τ_1_ (ms)	τ_2_ (ms)	<τ> (ms)
294	315/543.5	0.10 ± 0.01	0.40 ± 0.01	0.35
12	315/543.5	0.09 ± 0.01	0.38 ± 0.01	0.33

## Data Availability

The data supporting this study’s findings are available from the corresponding author upon reasonable request.

## Supplementary Materials

The following supporting information can be downloaded at: https://www.mdpi.com/article/10.3390/nano12244380/s1, Table S1: Elemental analysis of Tb-MOF, compound **1**; Table S2: Crystallographic data and structure refinement details of compound **1**; Table S3: Selected bond lengths (Å) and angles (°) for compound **1**; Table S4: Table of the continuous Shape Measurements for the TbN_3_O_6_ coordination environment; Table S5: Table of the continuous Shape Measurements for the TbO_8_ coordination environment; Figure S1: Schematic representation of how pellets preparations have been performed with the home-made extrusion apparatus and how the coating of the pellets was carried out (up) and picture of an uncoated pellet of compound **1** (down); Figure S2: Schematic representation of how membrane preparations have been performed (up) and picture compound **1** immobilized in polymethyl methacrylate (PMMA) and polysulphone (PSF) membranes from left to right, respectively (down); Figure S3: Cross section EDS mapping of **1@PMMA** (up) and **1@PSF** (button) membranes; Figure S4. Diffuse reflectance of compound **1** (black solid line), **1@PMMA** (red solid line) and pristine PMMA membrane (dashed red); Table S6: Comparison of lifetime values of compound **1** in bulk and PMMA membrane at ambient temperature (294 K) and low temperature (12 K); Figure S5: Ambient temperature micro-photoluminescence images taken on single-crystal of compound **1** at different excitation lines; Figure S6: Nyquist plot for compound **1** collected at 60 °C under variable RH (V_ac_ = 0.1 V). The numbers indicate the peak frequency; Table S7: Conductivity of 3D carboxylate-based MOFs. References [28,29,30,31,32,33,34,35,36,37,40] are cited in the supplementary materials.
