# Supplementary material for: Easy Handling and Cost-Efficient Processing of a Tb3+-MOF: The Emissive Capacity of the Membrane-Immobilized Material, Water Vapour Adsorption and Proton Conductivity
Source: Nanomaterials (Basel). 2022 Dec 8;12(24):4380. doi: 10.3390/nano12244380 (PMC9784928; doi:10.3390/nano12244380)
Supplement: Supplementary file 1 [file nanomaterials-12-04380-s001.zip › nanomaterials-2032994-supplementary-v2.pdf]

nanomaterials

Supporting Information

Easy Handling and Cost-Efficient Processing of a Tb^{3+}-MOF: The Emissive Capacity of the Membrane-Immobilized Material, Water Vapour Adsorption and Proton Conductivity

Estitxu Echenique-Errandonea [1], Ricardo Faria Mendes [2], Flávio Figueira [2], Paula Barbosa [3], Sara Rojas [4], Duane Choquesillo-Lazarte [5], Javier Cepeda [1], Duarte Ananias [2], Filipe Figueiredo [3], Filipe A. Almeida Paz [2], Antonio Rodríguez-Diéguez [4,*] and José Manuel Seco [1,*]

1 Departamento de Química Aplicada, Facultad de Química, Universidad del País Vasco UPV/EHU, Paseo Manuel Lardizabal, Nº 3, 20018 Donostia-San Sebastián, Spain
2 Department of Chemistry, CICECO–Aveiro Institute of Materials, University of Aveiro, 3810-193 Aveiro, Portugal
3 Department of Physics, CICECO–Aveiro Institute of Materials, University of Aveiro, 3810-193 Aveiro, Portugal
4 Departamento de Química Inorgánica, Facultad de Ciencias, Universidad de Granada, Av. Fuentenueva S/N, 18071 Granada, Spain
5 Laboratorio de Estudios Cristalográficos, IACT, CSIC-UGR, Av. Las Palmeras Nº 4, Armilla, 18100 Granada, Spain
* Correspondence: antonio5@ugr.es (A.R.-D.); josemanuel.seco@ehu.eus (J.M.S.)

Table of Contents:

Experimental Section S1
General Instrumentation S1
Chemical Characterization of Compounds S3
Elemental Analysis S3
Crystallographic Data S3
Selected Bond Lengths and Angles Data S4
Continuous Shape Measurements S6
Transformation Into Pellets and Membranes S7
Scanning Electron Microscopy S8
Photoluminescence Measurements S9
Electrical Conductivity S10
References S11

S1. Experimental Section

S1.1. General Instrumentation

X-ray powder diffraction (XRPD) patterns were collected at 25 °C on a Phillips X'PERT powder diffractometer with Cu-Kα radiation (λ = 1.5418 Å) over the range 5 < 2θ < 50° with a step size of 0.02° and an acquisition time of 2.5 s per step. Indexation of the diffraction profiles were made by means of the FULLPROF program (pattern- matching

analysis) based on the space group and the cell parameters found by single crystal X-ray diffraction.[1]

Scanning electron microscopy (SEM) images were acquired using either a Hitachi S4100 field emission gun tungsten filament instrument working at 25 kV or a high-resolution Hitachi SU-70 working at 4 kV. Samples were prepared by deposition on aluminium sample holders followed by carbon coating using an Emitech K950X carbon evaporator. EDS (energy dispersive X-ray spectroscopy) data and SEM mapping images were recorded using the latter microscope working at 15 kV and using either a Bruker Quantax 400 or an Esprit 1.9 EDS microanalysis system.

Dynamic water vapour sorption (DVS) measurements were performed on a DVS Resolution water vapour analyzer (Surface Measurement Systems™), at 25 °C and from 0 to 95% RH, with steps of 10% RH, using 200 SCCM N2 (N50, purity ≥ 99.999%) as carrier gas. Both the sorption and desorption curves were recorded by setting a stability criterion for the mass change (gravimetric precision of 0.1 μg) as the minimum in the variation of the mass over the time variation (dm/dt) of 0.002 % min^{-1}, or a maximum stage time at each RH of 360 min (in the case when the dm/dt minimum was not attained). Prior to the measurements, the sample of **1** was pretreated in situ in at 75 °C for 2 h at 0% RH and then at 25 °C for 1h at 0% RH, to ensure dehumidification for an initial reference state. An equivalent treatment was used for samples **1@PSF** and **1@PMMA** but with the first isothermal dwell at 60 °C to prevent softening of the polymer matrices.

The electrical conductivity (σ) of pelletized sample **1** and composite membranes (**1@PSF** and **1@PMMA**) were studied by impedance spectroscopy using an Agilent E4980A Precision LCR meter. Disc shaped pure sample **1** was obtained after pressing the powder in a uniaxial press at 853 MPa. Silver electrodes were applied on both sides of the pellet, 1@PSF and 1@PMMA (with an area of approximately 1x1 cm^2) by painting a commercial paste (Agar Scientific). The electrical resistance of pristine PSF membrane was measured using an in-plane configuration. The electrodes were applied on a rectangular membrane (~1 × 0.5 cm^2) by painting two stripes of a silver paste (Agar Scientific) separated by ~1 cm. Samples were placed on ceramic tubular sample holders (equipped with platinum wires for current collection) inside a climatic chamber (ACS DY110) in order to carry out the measurements under variable temperature (40–94 °C) and relative humidity (RH, 20–95%). The impedance spectra were collected between 20 Hz and 2 MHz with a test signal amplitude of 100 mV. The current collection was ensured by separate platinum wires for voltage and current. The spectra were analyzed with ZView (Version 2.6b, Scribner Associates) to assess the ohmic resistance (R), which was then normalized to the samples geometry to calculate the conductivity using Eq. 1:

$$\sigma = L(RA)^{-1} \tag{1}$$

where L is the sample thickness and A is the surface area of the electrodes. The maximum relative error in the conductivity data is estimated to be of the order of 4.6% for compound **1** and 2.5% for **1@PSF** membrane through the conventional chain rule of differentiation of Equation (X) and the uncertainties in the measured parameters (ΔR = 0.1% to 0.3% of R, $\Delta L = \pm 0.002\ cm, \Delta A = \pm\ 0.005\ cm$).

Photoluminescence Spectroscopy. The emission and excitation spectra were recorded at ambient-temperature and 12 K using a Fluorolog®-3Horiba Scientific (Model FL3-2T) spectroscope, with a modular double grating excitation spectrometer (fitted with a 1200 grooves/mm grating blazed at 330 nm) and a TRIAX 320 single emission monochromator (fitted with a 1200 grooves/mm grating blazed at 500 nm, reciprocal linear density of 2.6 $nm{\cdot}mm^{-1}$), coupled to a R928 Hamamatsu photomultiplier, using the front face acquisition mode. The excitation source was a 450 W Xe arc lamp. The emission spectra were corrected for detection and optical spectral response of the spectrofluorimeter and the excitation spectra were corrected for the spectral distribution of the lamp intensity using a photodiode reference detector. Time-resolved measurements have been carried out using a 1934D3 phosphorimeter coupled to the Fluorolog®-3, and a Xe-Hg flash lamp (6 μs/pulse

half width and 20–30 μs tail) was used as the excitation source. The low temperature measurements (12 K) were performed using a helium-closed cycle cryostat with vacuum system measuring ca. 5 × 10^{-6} mbar and a Lakeshore 330 auto-tuning temperature controller with a resistance heater.

S2. Chemical Characterization of Compounds

S2.1. Elemental Analysis

Table S1. Elemental analysis of Tb-MOF, compound 1.

Compound	Formula	Molecular weight	Calc.	Found.
**1**	$C_{57}H_{74}N_{11}O_{29}Tb_5$	2171.9	C: 31.52; H: 3.43; N: 7.09; O: 21.36; Tb: 36.59	C: 31.56; H: 3.40; N: 7.11; O: 21.42; Tb: 36.61

S3. Crystallographic Data

Table S2. Crystallographic data and structure refinement details of compound 1.

Compound	1
Formula	$C_{57}H_{74}N_{11}O_{29}Tb_5$
M_r	2171.87
CCDC number	2195455
Crystal system	hexagonal
Space group (no.)	*P*63/*m* (176)
a(Å)	15.8519(6)
b(Å)	15.8519(6)
c(Å)	16.8695(11)
α(°)	90
β(°)	90
γ(°)	120
V(Å^3)	3671.1 (4)
Z	2
ρ_{calc}g/cm^3	1.965
μ/mm^{-1}	4.841
F(000)	2100.0
Crystal size/mm^3	0.625 × 0.171 × 0.092
Radiation	MoKα (λ = 0.71073)
2Θ range for data collection/°	5.14 to 54.944
Index ranges	−20 ≤ h ≤ 20, −17 ≤ k ≤ 20, −21 ≤ l ≤ 21
Reflections collected	36639
Independent reflections	2905 [R_{int} = 0.0356, R_{sigma} = 0.0142]
Data/restraints/parameters	2905/0/123
Goodness-of-fit on F^2	1.083
Final R indexes [I>=2σ (I)]	R_1 = 0.0148, wR_2 = 0.0392
Final R indexes [all data]	R_1 = 0.0165, wR_2 = 0.0398
Largest diff. peak/hole / e Å^{-3}	0.71/−0.50

S4. Selected Bond Lengths and Angles Data

Table S3. Selected bond lengths (Å) and angles (°) for compound 1.

Atom	Atom	Length/Å
Tb1	$Tb1^{1}$	3.5426(4)
Tb1	$O1^{2}$	2.5154(15)
Tb1	$O1^{3}$	2.5154(15)
Tb1	O1	2.5154(15)
Tb1	$N1^{3}$	2.5148(18)
Tb1	$N1^{2}$	2.5148(18)
Tb1	N1	2.5148(18)
Tb1	$Tb2^{2}$	3.90972(19)
Tb1	Tb2	3.90975(19)
Tb1	$O4^{3}$	2.3861(13)
Tb1	O4	2.3861(14)
Tb1	$O4^{2}$	2.3861(13)
Tb2	O1	2.3509(14)
Tb2	$O2^{4}$	2.4267(15)
Tb2	$O2^{5}$	2.4267(15)
Tb2	$O3^{5}$	2.4396(15)
Tb2	$O3^{4}$	2.4396(15)
Tb2	O4	2.326(2)
Tb2	O5	2.359(2)

^{1} +x,+y,3/2−z; ^{2} 1+y−x,1−x,+z; ^{3} 1−y,+x−y,+z; ^{4} 1−y+x,+x,1−z; ^{5} 1−y+x,+x,1/2+z

Table S3. Selected bond lengths (Å) and angles (°) for compound 1. (continuation).

Atom	Atom	Atom	Angle/˚	Atom	Atom	Atom	Angle/˚
N1	Tb1	$Tb1^{1}$	131.70(5)	$O1^{1}$	Tb2	$Tb1^{1}$	38.00(4)
N1	Tb1	$O1^{3}$	69.05(5)	$O1^{1}$	Tb2	Tb1	85.08(4)
N1	Tb1	O1	65.47(5)	$O1^{1}$	Tb2	O1	101.63(7)
N1	Tb1	$O1^{2}$	137.07(6)	$O1^{1}$	Tb2	$O2^{4}$	82.06(5)
N1	Tb1	Tb2	100.27(4)	$O1^{1}$	Tb2	$O2^{5}$	145.54(5)
N1	Tb1	$Tb2^{2}$	158.35(4)	$O1^{1}$	Tb2	$O3^{4}$	86.92(5)
$N1^{2}$	Tb1	$Tb1^{1}$	131.70(5)	$O1^{1}$	Tb2	$O3^{5}$	159.28(5)
$N1^{2}$	Tb1	$O1^{3}$	137.07(6)	$O1^{1}$	Tb2	O5	79.84(5)
$N1^{2}$	Tb1	O1	69.05(5)	$O1^{2}$	Tb1	$Tb1^{1}$	91.16(3)
$N1^{2}$	Tb1	$O1^{2}$	65.47(5)	$O1^{2}$	Tb1	$O1^{3}$	119.959(3)
$N1^{2}$	Tb1	N1	80.57(7)	$O1^{2}$	Tb1	O1	119.958(3)
$N1^{2}$	Tb1	$Tb2^{2}$	100.27(4)	$O1^{2}$	Tb1	$Tb2^{2}$	35.13(3)
$N1^{2}$	Tb1	Tb2	78.26(5)	$O1^{2}$	Tb1	Tb2	97.75(3)
$N1^{3}$	Tb1	$Tb1^{1}$	131.70(5)	$O1^{3}$	Tb1	$Tb1^{1}$	91.16(3)
$N1^{3}$	Tb1	$O1^{2}$	69.05(5)	$O1^{3}$	Tb1	O1	119.960(2)
$N1^{3}$	Tb1	$O1^{3}$	65.47(5)	$O1^{3}$	Tb1	Tb2	135.28(3)
$N1^{3}$	Tb1	O1	137.07(6)	$O1^{3}$	Tb1	$Tb2^{2}$	97.75(3)
$N1^{3}$	Tb1	N1	80.57(7)	$O2^{4}$	Tb2	Tb1	109.80(4)
$N1^{3}$	Tb1	$N1^{2}$	80.57(7)	$O2^{4}$	Tb2	$Tb1^{1}$	77.06(4)
$N1^{3}$	Tb1	$Tb2^{2}$	78.26(5)	$O2^{4}$	Tb2	$O2^{5}$	76.78(8)
$N1^{3}$	Tb1	Tb2	158.35(4)	$O2^{4}$	Tb2	$O3^{4}$	53.79(5)
O1	Tb1	$Tb1^{1}$	91.16(3)	$O2^{4}$	Tb2	$O3^{5}$	101.63(5)
O1	Tb1	Tb2	35.13(3)	$O2^{5}$	Tb2	Tb1	77.06(4)

O1	Tb1	Tb2^{2}	135.28(3)	O2^{5}	Tb2	Tb1^{1}	109.80(4)
O1	Tb2	Tb1^{1}	85.09(4)	O2^{5}	Tb2	O3^{4}	101.63(5)
O1	Tb2	Tb1	38.00(4)	O2^{5}	Tb2	O3^{5}	53.79(5)
O1	Tb2	O2^{4}	145.53(5)	O3^{4}	Tb2	Tb1^{1}	112.13(4)
O1	Tb2	O2^{5}	82.06(5)	O3^{4}	Tb2	Tb1	162.71(4)
O1	Tb2	O3^{4}	159.28(5)	O3^{4}	Tb2	O3^{5}	79.29(7)
O1	Tb2	O3^{5}	86.91(5)	O3^{5}	Tb2	Tb1^{1}	162.72(4)
O1	Tb2	O5	79.84(5)	O3^{5}	Tb2	Tb1	112.13(4)

^{1}+x,+y,3/2-z; ^{2}1+y-x,1-x,+z; ^{3}1-y,+x-y,+z; ^{4}1-y+x,+x,1/2+z; ^{5}1-y+x,+x,1-z; ^{6}+y,1-x+y,1-z

Table S3. Selected bond lengths (Å) and angles (°) for compound 1. (continuation).

Atom	Atom	Atom	Angle/°	Atom	Atom	Atom	Angle/°
O4	Tb1	Tb1^{1}	42.07(4)	O4^{3}	Tb1	O1^{3}	68.56(6)
O4	Tb1	O1^{3}	133.06(5)	O4^{3}	Tb1	O1^{2}	133.06(5)
O4	Tb1	O1^{2}	74.21(6)	O4^{3}	Tb1	O1	74.21(6)
O4	Tb1	O1	68.56(6)	O4^{3}	Tb1	N1	89.86(6)
O4	Tb1	N1^{2}	89.86(6)	O4^{3}	Tb1	N1^{2}	142.76(6)
O4	Tb1	N1^{3}	142.76(6)	O4^{3}	Tb1	N1^{3}	133.47(6)
O4	Tb1	N1	133.47(6)	O4^{3}	Tb1	Tb2	68.12(5)
O4	Tb1	Tb2^{2}	68.12(5)	O4^{3}	Tb1	Tb2^{2}	101.43(4)
O4	Tb1	Tb2	33.44(5)	O5	Tb2	Tb1	110.33(5)
O4	Tb1	O4^{3}	70.94(6)	O5	Tb2	Tb1^{1}	110.33(5)
O4	Tb2	Tb1^{1}	34.42(3)	O5	Tb2	O2^{5}	133.97(5)
O4	Tb2	Tb1	34.42(3)	O5	Tb2	O2^{4}	133.97(5)
O4	Tb2	O1^{1}	72.41(5)	O5	Tb2	O3^{4}	83.20(6)
O4	Tb2	O1	72.41(5)	O5	Tb2	O3^{5}	83.20(6)
O4	Tb2	O2^{5}	76.31(5)	Tb1	Tb2	Tb1^{1}	53.878(6)
O4	Tb2	O2^{4}	76.31(5)	Tb1	O4	Tb1^{1}	95.86(7)
O4	Tb2	O3^{5}	128.31(5)	Tb1^{1}	Tb1	Tb2^{2}	63.060(3)
O4	Tb2	O3^{4}	128.31(5)	Tb1^{1}	Tb1	Tb2	63.061(3)
O4	Tb2	O5	135.22(8)	Tb2	O1	Tb1	106.87(5)
O4^{2}	Tb1	Tb1^{1}	42.07(4)	Tb2	O4	Tb1	112.14(6)
O4^{2}	Tb1	O1^{2}	68.56(6)	Tb2	O4	Tb1^{1}	112.14(6)
O4^{2}	Tb1	O1^{3}	74.21(6)	Tb2^{2}	Tb1	Tb2	101.077(4)
O4^{2}	Tb1	O1	133.06(5)				
O4^{2}	Tb1	N1	142.76(6)				
O4^{2}	Tb1	N1^{3}	89.86(6)				
O4^{2}	Tb1	N1^{2}	133.47(6)				
O4^{2}	Tb1	Tb2^{2}	33.44(5)				
O4^{2}	Tb1	Tb2	101.43(4)				
O4^{2}	Tb1	O4^{3}	70.94(6)				
O4^{2}	Tb1	O4	70.94(6)				
O4^{3}	Tb1	Tb1^{1}	42.07(4)				

^{1}+x,+y,3/2−z; ^{2}1+y−x,1−x,+z; ^{3}1−y,+x−y,+z; ^{4}1−y+x,+x,1/2+z; ^{5}1−y+x,+x,1−z; ^{6}+y,1−x+y,1−z

S4. Continuous Shape Measurements

Table S4. Table of the continuous Shape Measurements for the TbN_3O_6 coordination environment.

EP-9	1 D9h	Enneagon
OPY-9	2 C8v	Octagonal pyramid
HBPY-9	3 D7h	Heptagonal bipyramid
JTC-9	4 C3v	Johnson triangular cupola J3
JCCU-9	5 C4v	Capped cube J8
CCU-9	6 C4v	Spherical-relaxed capped cube
JCSAPR-9	7 C4v	Capped square antiprism J10
CSAPR-9	8 C4v	Spherical capped square antiprism
JTCTPR-9	9 D3h	Tricapped trigonal prism J51
TCTPR-9	10 D3h	Spherical tricapped trigonal prism
JTDIC-9	11 C3v	Tridiminished icosahedron J63
HH-9	12 C2v	Hula-hoop
MFF-9	13 Cs	Muffin

Complex	JCSAPR-9	CSAPR-9	JTCTPR-9	TCTPR-9	MFF-9
**Tb1**	2.333	1.355	1.982	**0.629**	2.038

Table S5. Table of the continuous Shape Measurements for the TbO_8 coordination environment.

OP-8	1 D8h	Octagon
HPY-8	2 C7v	Heptagonal pyramid
HBPY-8	3 D6h	Hexagonal bipyramid
CU-8	4 Oh	Cube
SAPR-8	5 D4d	Square antiprism
TDD-8	6 D2d	Triangular dodecahedron
JGBF-8	7 D2d	Johnson - Gyrobifastigium (J26)
JETBPY-8	8 D3h	Johnson - Elongated triangular bipyramid (J14)
JBTP-8	9 C2v	Johnson - Biaugmented trigonal prism (J50)
BTPR-8	10 C2v	Biaugmented trigonal prism
JSD-8	11 D2d	Snub disphenoid (J84)
TT-8	12 Td	Triakis tetrahedron
ETBPY-8	13 D3h	Elongated trigonal bipyramid

Complex	SAPR-8	TDD-8	JBTPR-8	BTPR-8	JSD-8
**Tb2**	**2.686**	2.698	3.641	3.239	5.494

S5. Transformation Into Pellets and Membranes

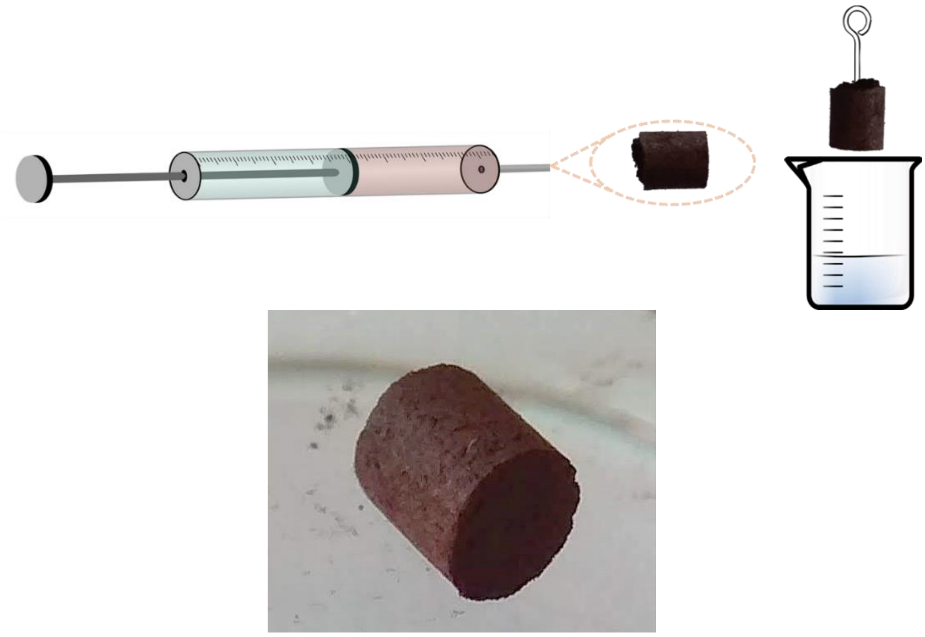

Figure S1. Schematic representation of how pellets preparations have been performed with the home-made extrusion apparatus and how the coating of the pellets was done (up) and picture of an uncoated pellet of compound 1 (down).

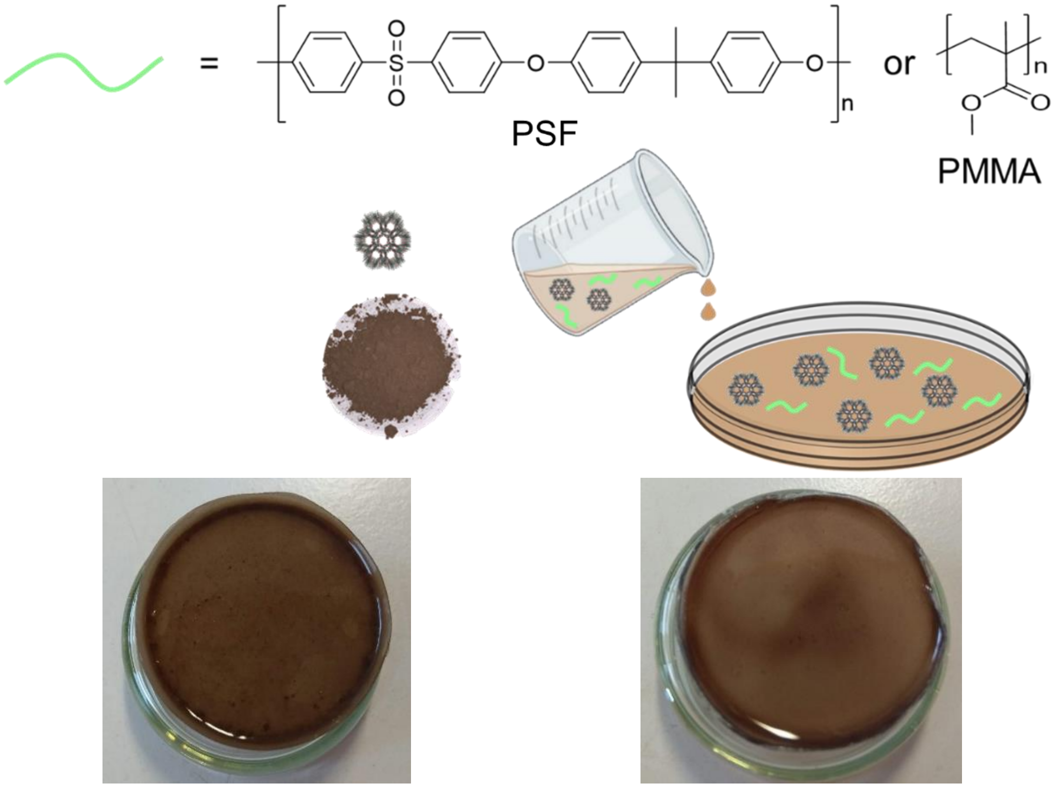

Figure S2. Schematic representation of how membrane preparations has been performed (up) and picture compound 1 immobilized in polymethyl methacrylate (PMMA) and polysulphone (PSF) membranes from left to right, respectively (down).

S6. Scanning Electron Microscopy

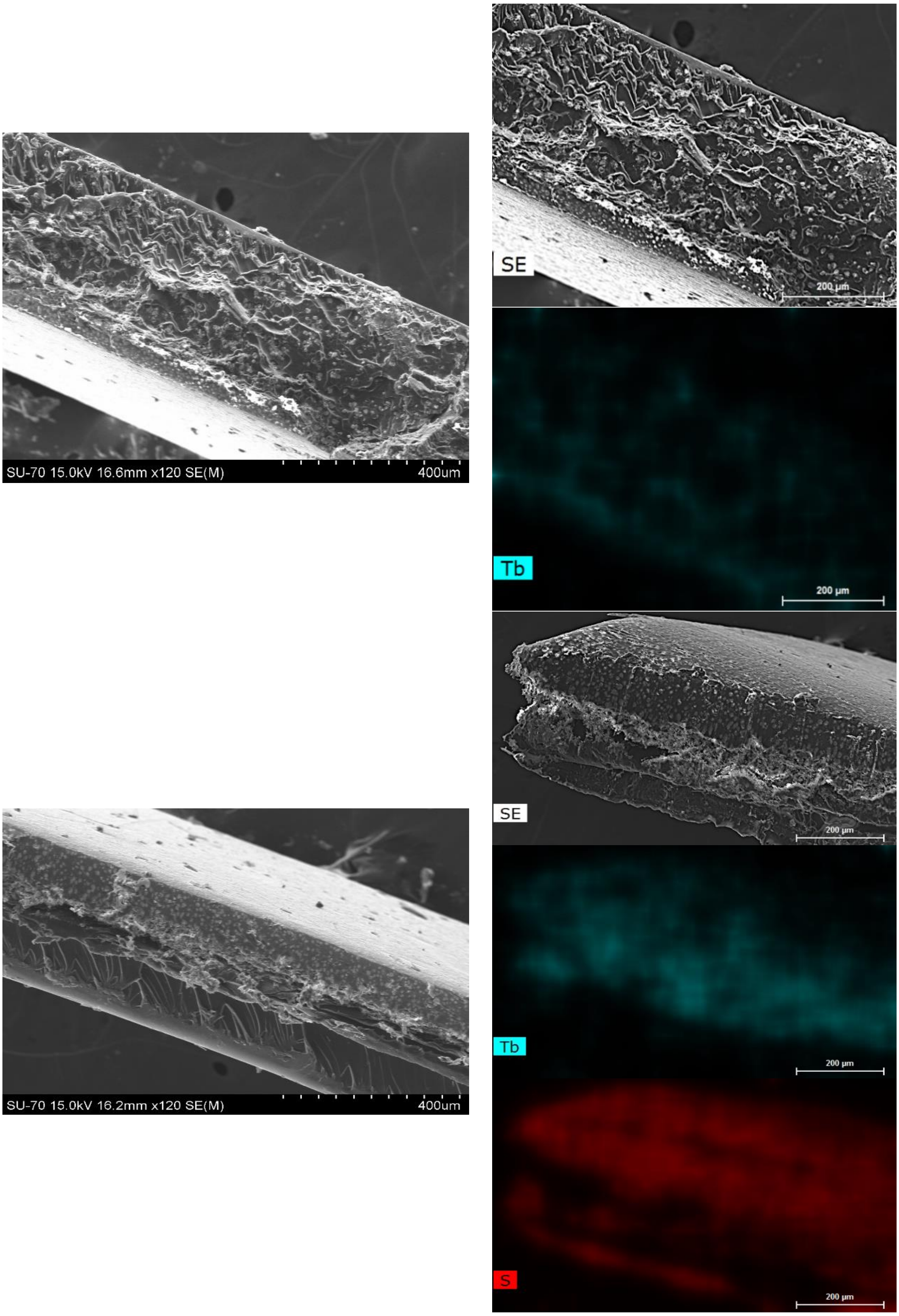

Figure S3. Cross section EDS mapping of **1@PMMA** (up) and **1@PSF** (bottom) membranes.

S7. Diffuse Reflectance Measurements

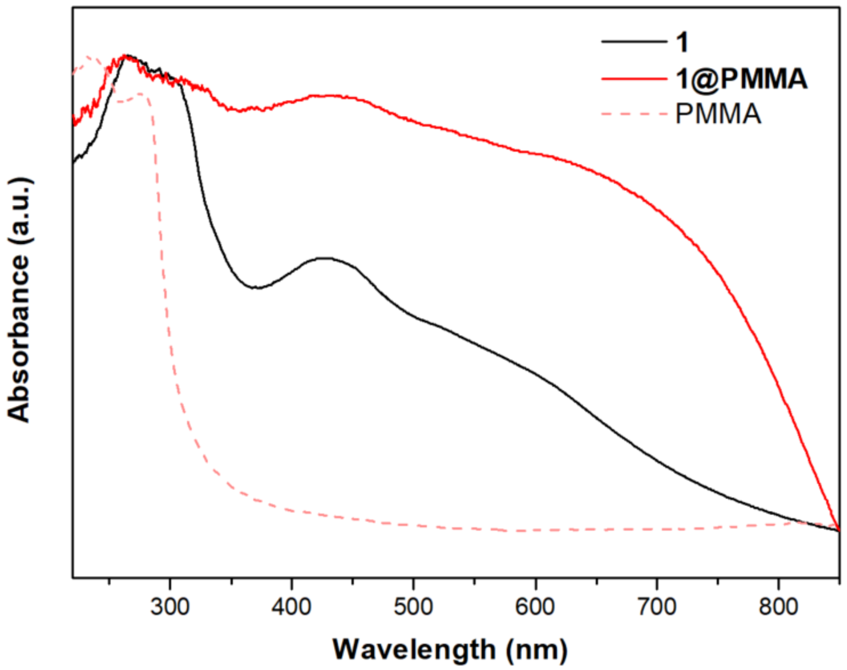

Figure S4. Diffuse reflectance of compound 1 (black solid line), 1@PMMA (red solid line) and pristine PMMA membrane (dashed red).

S8. Photoluminescence Measurements

Table S6. Comparison of lifetime values of compound 1 in bulk and PMMA membrane at ambient temperature (294 K) and low temperature (12 K).

Compound	τ_1 / ms	τ_2/ ms	<τ> / ms
**1 (377)**	0.08 ± 0.01	0.36 ± 0.01	0.29
**1@PMMA at 294 K (315)**	0.10 ± 0.01	0.40 ± 0.01	0.35
**1@PMMA at 12 K (315)**	0.09 ± 0.01	0.38 ± 0.01	0.33

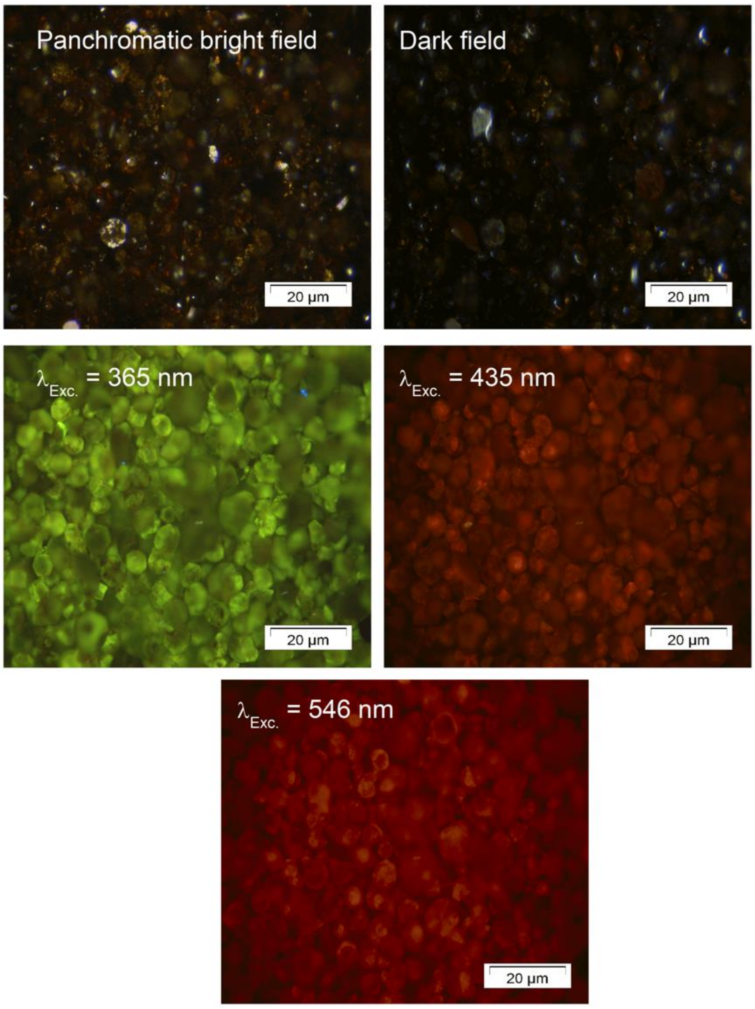

Figure S5. Ambient temperature micro-photoluminescence images taken on single-crystal of compound 1 at different excitation lines.

S9. Electrical Conductivity

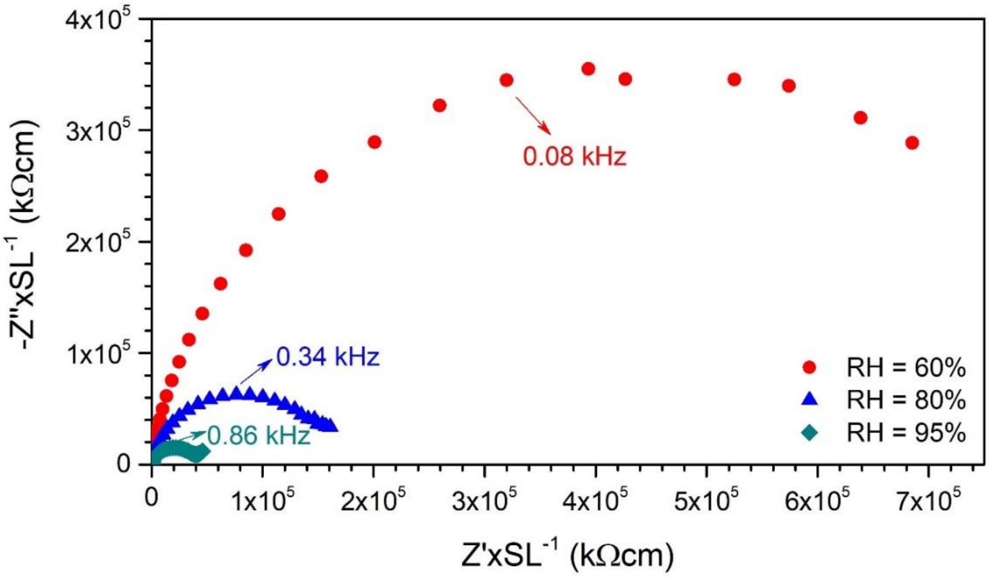

Figure S6. Nyquist plot for compound **1** collected at 60 ºC under variable RH (V_{ac} = 0.1 V). The numbers indicate the peak frequency.

Table S7. Conductivity of 3D carboxylate-based MOFs.

MOF	Conductivity (S cm^{-1})	Reference
{H[(N(CH$_3$)$_4$)$_2$][Gd$_3$(NIPA)$_6$]}·3H$_2$O	7.17 × 10^{-2} (75 °C, 98 % RH)	[2]
[Gd$_4$(*di*-nitro-BPDC)$_4$(NO$_2$)$_3$(OH)(H$_2$O)$_5$]·(solvent)	5.76 × 10^{-2} (55 °C, 99 % RH)	[3]
[In(EBTC)(CH$_3$)$_2$NH$_2$]·DMF·(H$_2$O)$_5$	3.49 × 10^{-3} (25 °C, 99 % RH)	[4]
{[M$_2$Cl$_2$(BTC)$_{4/3}$]·(Me$_2$NH$_2$)$^+{}_2$·4/3H$_2$O}$_n$ (M = Co (1) and Mn (2))	1.19 × 10^{-3} (50 °C, 65 % RH) (1)<br>2.60 × 10^{-4} (19 °C, 65 % RH) (2)	[5]
MOF-808-OX<br>MOF-808-EDTA	4.25 × 10^{-4} (80 °C, 98 % RH)<br>1.31 × 10^{-4} (30 °C, 98 % RH)	[6]
[Bi$_4$(HAzoBTC)$_2$(AzoBTC)(OH)$_2$(H$_2$O)$_4$]·7H$_2$O	1.1 × 10^{-4} (80 °C, 80 % RH)	[7]
Fe–MOF<br>Im@Fe–MOF<br>Im–Fe–MOF	1.25 × 10^{-4} (60 °C, 98% RH)<br>4.23 × 10^{-3} (60 °C, 98 % RH)<br>1.21 × 10^{-2} (60 °C, 98 % RH)	[8]
[Cd$_2$(btc)$_2$(H$_2$O)$_2$]$_n$·*n*(H$_2$bmib)·6*n*(H$_2$O)<br>[Cd$_4$(cpip)$_2$(Hcpip)$_2$]$_n$·*n*(H$_2$bmib)·n(H$_2$O)	5.4 × 10^{-5} (60 °C, 95 % RH)<br>2.2 × 10^{-5} (60 °C, 95 % RH)	[9]
MFM-510 [Ba$_2$(L^1)(H$_2$O)$_{1.5}$(CO$_2$)(DMF)$_{1.5}$]<br>MFM-511 [Ba(H$_2$L^2)(H$_2$O)(DMF)]<br>MFM-512 [Ba$_2$(HL^3)(H$_2$O)$_4$]	2.1 × 10^{-5} (25 °C, 99 % RH)<br>5.1 × 10^{-5} (25 °C, 99 % RH)<br>2.9 × 10^{-3} (25 °C, 99 % RH)	[10]
[Cd$_5$(TCA)$_2$(H$_2$O)$_2$] 8DMA·16H$_2$O	1.45 × 10^{-6} (80 °C, 85 % RH)	[11]

References

1. Rodríguez-Carvajal, J. *FULLPROF 2000*, version 2.5d; Lab. Léon Brillouin (CEA-CNRS), Cent. d'Études Saclay: Gif sur Yvette, France, 2000.
2. Xing, X.-S.; Fu, Z.-H.; Zhang, N.-N.; Yu, X.-Q.; Wang, M.-S.; Guo, G.-C. High proton conduction in an excellent water-stable gadolinium metal–organic framework. *Chem. Commun.* **2019**, *55*, 1241–1244, https://doi.org/10.1039/c8cc08700h.
3. Thammakan, S.; Rodlamul, P.; Semakul, N.; Yoshinari, N.; Konno, T.; Ngamjarurojana, A.; Rujiwatra, A. Gas Adsorption, Proton Conductivity, and Sensing Potential of a Nanoporous Gadolinium Coordination Framework. *Inorg. Chem.* **2020**, *59*, 3053–3061, https://doi.org/10.1021/acs.inorgchem.9b03395.
4. Zhai, L.; Yu, J.-W.; Zhang, J.; Zhang, W.-W.; Wang, L.; Ren, X.-M. High quantum yield pure blue emission and fast proton conduction from an indium–metal–organic framework. *Dalton Trans.* **2019**, *48*, 12088–12095, https://doi.org/10.1039/c9dt02472g.
5. Liu, S.-J.; Cao, C.; Yang, F.; Yu, M.-H.; Yao, S.-L.; Zheng, T.-F.; He, W.-W.; Zhao, H.-X.; Hu, T.-L.; Bu, X.-H. High Proton Conduction in Two Co^{II} and Mn^{II} Anionic Metal–Organic Frameworks Derived from 1,3,5-Benzenetricarboxylic Acid. *Cryst. Growth Des.* **2016**, *16*, 6776–6780, https://doi.org/10.1021/acs.cgd.6b00776.
6. Meng, X.; Wang, H.-N.; Wang, L.-S.; Zou, Y.-H.; Zhou, Z.-Y. Enhanced proton conductivity of a MOF-808 framework through anchoring organic acids to the zirconium clusters by post-synthetic modification. *CrystEngComm* **2019**, *21*, 3146–3150, https://doi.org/10.1039/c9ce00328b.
7. Vilela, S.M.F.; Devic, T.; Varez, A.; Salles, F.; Horcajada, P. A new proton-conducting Bi-carboxylate framework. *Dalton Trans.* **2019**, *48*, 11181–11185, https://doi.org/10.1039/c9dt02009h.
8. Zhang, F.-M.; Dong, L.-Z.; Qin, J.-S.; Guan, W.; Liu, J.; Li, S.-L.; Lu, M.; Lan, Y.-Q.; Su, Z.-M.; Zhou, H.-C. Effect of Imidazole Arrangements on Proton-Conductivity in Metal–Organic Frameworks. *J. Am. Chem. Soc.* **2017**, *139*, 6183–6189, https://doi.org/10.1021/jacs.7b01559.
9. Li, X.; Sun, X.; Li, X.; Fu, Z.; Su, Y.; Xu, G. Porous Cadmium(II) Anionic Metal–Organic Frameworks Based on Aromatic Tricarboxylate Ligands: Encapsulation of Protonated Flexible Bis(2-methylimidazolyl) Ligands and Proton Conductivity. *Cryst. Growth Des.* **2015**, *15*, 4543–4548, https://doi.org/10.1021/acs.cgd.5b00799.
10. Rought, P.; Marsh, C.; Pili, S.; Silverwood, I.P.; Sakai, V.G.; Li, M.; Brown, M.S.; Argent, S.P.; Vitorica-Yrezabal, I.; Whitehead, G.; et al. Modulating proton diffusion and conductivity in metal–organic frameworks by incorporation of accessible free carboxylic acid groups. *Chem. Sci.* **2018**, *10*, 1492–1499, https://doi.org/10.1039/c8sc03022g.
11. Shen, Y.; Yang, X.-F.; Zhu, H.-B.; Zhao, Y.; Li, W.-S. A unique 3D metal–organic framework based on a 12-connected pentanuclear Cd(ii) cluster exhibiting proton conduction. *Dalton Trans.* **2015**, *44*, 14741–14746, https://doi.org/10.1039/c5dt02544c.
